# Recombinant subunit vaccine against *Mycoplasma gallisepticum* disease confers cross-protection against multiple pathogenic strains

**DOI:** 10.1128/iai.00025-26

**Published:** 2026-06-12

**Authors:** Jeremy M. Miller, Rosemary G. Ozyck, Arlind B. Mara, Esmeralda F. Hernandez, Jessica B. Malek, Edan R. Tulman, Steven M. Szczepanek, Steven J. Geary

**Affiliations:** 1 Department of Pathobiology, University of Connecticut 7712, Storrs, Connecticut, USA; 2 Center of Excellence for Vaccine Research, University of Connecticut 7712, Storrs, Connecticut, USA; 3 US Animal Vaccinology Research Coordination Network, Storrs, Connecticut, USA; 4 Department of Allied Health Sciences, University of Connecticut 248510, Storrs, Connecticut, USA; Rutgers New Jersey Medical School, Newark, New Jersey, USA

**Keywords:** recombinant, adaptive immune response, subunit, vaccine

## Abstract

*Mycoplasma gallisepticum* (MG) is an avian respiratory pathogen of global concern, responsible for hundreds of millions of dollars in estimated economic damage each year. Given that MG is distributed across every continent except Antarctica, any globally applicable vaccine must provide protection against numerous strains. We previously published that an R_low_-based subunit vaccine reduced both MG bacterial burden and tracheal pathology in a homologous infection model using virulent MG strain R_low_. It remains unknown if the subunit vaccine could reduce disease burden induced by heterologous MG strains. To test this, we utilized a prime-boost schedule and challenged with either strain VA94 or Ap3AS. The vaccine significantly reduced disease burden induced by both heterologous strains as indicated by reduced MG burden and by reduced tracheal pathology. This demonstrates that an MG subunit vaccine has global potential.

## INTRODUCTION

*Mycoplasma gallisepticum* (MG) is a respiratory pathogen of avian species with global economic concern (1). A global meta-analysis determined that the pooled occurrence of MG infection is 27% (2). Economic losses occur due to reduced egg laying, reduced hatchability, reduced feed conversion, and downgrading of carcasses. MG infection can lead to co-infections that contribute to the development of chronic respiratory disease (CRD). CRD is characterized by compromised function and integrity of the respiratory tract, and it can also compromise reproductive tract function (3). MG can be transmitted both horizontally and vertically. Horizontal transmission occurs primarily through aerosolized respiratory secretions, egg contact, and indirect transmission via fomites (4). Once infected, chickens may be considered infected for life (5).

In geographic regions, where MG control efforts are made, there are three main approaches: strict biosecurity, antibiotic use, and vaccination (6). Strict biosecurity has worked well in the United States based on the United States Department of Agriculture (USDA) National Poultry Improvement Plan (NPIP) for farmers that opt into the program (7, 8). However, control is hampered by global trade, wild bird populations, the potentially asymptomatic nature of the infection, and high prevalence rates (2, 9–11). Antibiotic use may help with MG control, but it does not always clear the infection, may lead to increased risk of antibiotic resistance, and results in economic loss as animals receiving antibiotic treatment must undergo a withdrawal period prior to their use for human consumption (12–15). To avoid issues associated with antibiotics, and for producers without the infrastructure or resources to maintain strict biosecurity, vaccination programs present a viable solution (16). Not classically considered for MG control is the diversity of MG strains across the globe, which may further complicate vaccine-based control efforts (17, 18).

To meet this need and address drawbacks of current vaccine designs, we previously developed a recombinant subunit vaccine against MG that provided significant protective efficacy against the disease. A subunit vaccine provides a consistent serologic response to identify vaccinated birds, which is not always the case with other vaccine designs (19, 20). It also provides a clear serologic distinction between vaccinated and infected animals. The vaccine retains a high safety profile with no risk of reversion to virulence or bacterial transformation. Furthermore, it remains highly and readily modifiable to combat new or highly variable strains.

Our subunit vaccine is based on MG strain R_low,_ and it incorporates six MG subunit components along with the adjuvant CpG ODN 2007. The six subunits encompass a soluble form of the primary adhesion, gallisepticum adhesion protein A (GapA), and a soluble form of an accessory adhesion, cytadhesin-related molecule A (CrmA). GapA and CrmA have been shown to be important for attachment, gliding motility, and virulence (21–24). The other four subunits are variable lipoprotein hemagglutinin As (VlhAs), specifically: VlhAs 3.03, 3.06, 4.07, and 5.05. Although there are approximately 40 paralogous and phase-variable *vlhA* genes present in MG strain R_low_, these specific 4 VlhAs are the gene products that cover the breadth of those expressed within the first 7 days *in vivo* in a tracheal infection model of the disease using strain R_low_ (25). Although VlhA’s function remains unconfirmed, hypotheses include the involvement in MG-host interaction, pathogenesis, and immune escape given their phase-variable nature and ability to generate strong antibody responses *in vivo* (26–28).

R_low_ was previously tested for its known virulence and frequent use in infection models (19, 25, 26, 29, 30). VA94 was chosen for comparison given its virulence in chickens, its historical significance, and its genomic divergence from R_low_ specific with regard to its VlhA profile (28, 31, 32). Ap3AS (a gift from Dr. Glenn Browning, University of Melbourne School of Veterinary Medicine) was chosen for its known pathogenicity in chickens, its frequent use in infection models, and its unique geographic emergence relative to R_low_ given that it was isolated from and is endemic to Australia (33–36).

Strain R_low_ was isolated by Dr. Henry Van Roekel from the University of Massachusetts before being propagated and characterized by Markham and Wong in 1952. It was either isolated from a turkey experiencing infectious sinusitis or a chicken experiencing chronic respiratory disease (37). Strain Ap3AS was first isolated by Dr. Kevin G. Whithear in Australia in 1989 from the air sacs of a broiler chicken displaying marked respiratory disease (33). Strain VA94 was first isolated from a house finch (*Carpodacus mexicanus*) in Virginia in 1994 after cases of conjunctivitis in house finches started appearing in several mid-Atlantic and eastern regions of the United States (31). This strain quickly spread through house finch populations of the eastern and later northern and western states. Although it does not cause a fatal disease in itself, it resulted in a significant die-off across the eastern United States due to an inability to find food and avoid predators (31, 38–42). This event highlighted the need to be prepared for an emergent strain, as if this type of situation were to occur in farmed avian populations, the economic consequences could be profound.

Although current MG vaccines are generally considered to have efficacy in the field, the effects of strain diversity and antigenic variation on MG vaccine efficacy are not well known. Given that our vaccine was designed using only six proteins from a specific strain, it was reasonable to hypothesize that its efficacy may not translate well to other MG strains. This is especially important to answer given that four of the six protein subunits are VlhAs, members of a paralogous family recognized as a potential source of genetic and phenotypic diversity among MG strains (28, 43). Given that efficacy against heterologous strains is desirable for MG vaccines, here we test our R_low_-based subunit vaccine against heterologous challenges associated with VA94 or Ap3AS.

## RESULTS

### Subunit vaccine components exhibit considerable amino acid identity across diverse MG strains that differ by strain and subunit

Blastp analysis of subunit vaccine components queried against strain VA94 or Ap3AS to identify protein similarities at the amino acid level. Identity varied by protein and strain (Table 1).

**TABLE 1:** BLAST-based protein sequence comparison between subunits in the vaccine and the most similar proteins from strains VA94 and Ap3AS

Query from R_low_ (native amino acids expressed)	Subject strain	Most similar protein ID	% Identity	% Query cover
GapA(36-999)	VA94	WP_014885899.1	93.54%	100%
CrmA(26-934)	VA94	WP_014885900.1	97.25%	100%
VlhA 3.03	VA94	WP_014886207.1	93.08%	100%
VlhA 3.06	VA94	WP_014886075.1	61.80%	100%
VlhA 4.07	VA94	WP_014886207.1	60.90%	100%
VlhA 5.05	VA94	WP_014886207.1	91.36%	100%
GapA(36-999)	Ap3AS	WP_318551551.1	93.33%	100%
CrmA(26-934)	Ap3AS	WP_065165154.1	97.03%	100%
VlhA 3.03	Ap3AS	WP_318551573.1	94.88%	100%
VlhA 3.06	Ap3AS	WP_318551129.1	84.91%	100%
VlhA 4.07	Ap3AS	WP_065170024.1	61.70%	100%
VlhA 5.05	Ap3AS	WP_065170024.1	93.02%	100%

### Subunit vaccine VlhAs exhibit moderate to no amino acid identity with VA94 *in-vivo*-expressed vlhAs

Blastp analysis of subunit vaccine VlhAs queried against strain VA94 *in-vivo*-expressed vlhAs at amino acid level. Subunit VlhAs show moderate sequence identity with either WP_014886378.1 or WP_014886209.1 but share almost no sequence identity with WP_014886072.1 (Table 2).

**TABLE 2:** Protein sequence comparison between VlhAs in the vaccine and dominant early-phase-expressed in-vivo vlhA expression from the tracheas of chickens infected with strain VA94

Query from R_low_	Expressed vlhA in VA94 protein ID	% Identity	% Query cover
VlhA 3.03	WP_014886072.1	61.36%	12%
VlhA 3.06	WP_014886072.1	100%	4%
VlhA 4.07	WP_014886072.1	97.14%	5%
VlhA 5.05	WP_014886072.1	61.36%	12%
VlhA 3.03	WP_014886378.1	43.69%	99%
VlhA 3.06	WP_014886378.1	47.75%	97%
VlhA 4.07	WP_014886378.1	55.95%	99%
VlhA 5.05	WP_014886378.1	43.39%	99%
VlhA 3.03	WP_014886209.1	43.35%	98%
VlhA 3.06	WP_014886209.1	48.08%	99%
VlhA 4.07	WP_014886209.1	56.47%	98%
VlhA 5.05	WP_014886209.1	42.46%	98%

### Subunit-vaccinated chickens recognize antigens from heterologous MG strains

**Fig 1:**
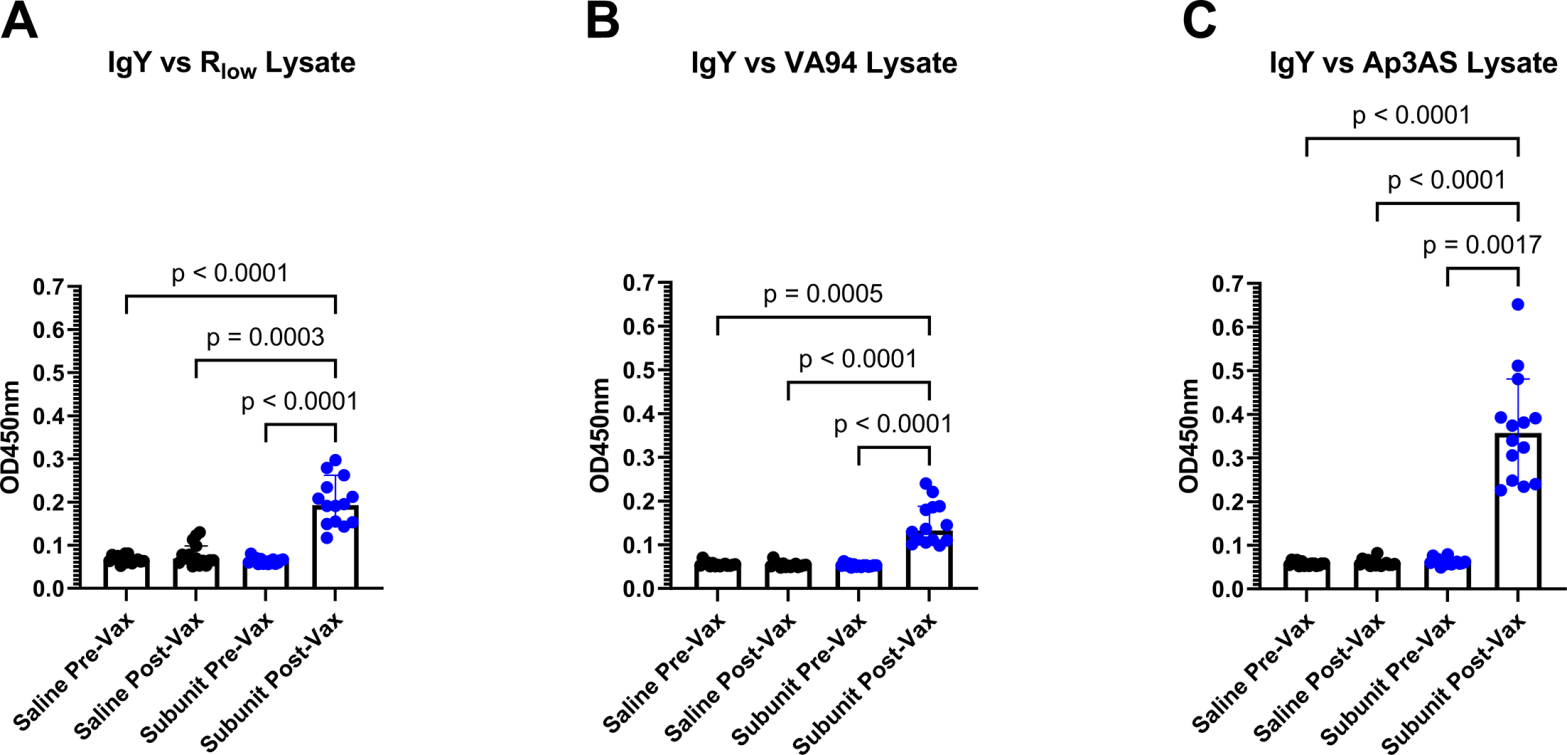
Subunit-vaccinated sera vs MG lysates. ELISAs run on sera diluted 1:1,000 from saline or subunit-vaccinated chickens taken prior to vaccination (Pre-Vax) or taken from chickens 3 weeks post-boost vaccination but prior to challenge (Post-Vax). Systemic IgY was assessed against R_low_ lysate (**A**), VA94 lysate (**B**), or Ap3AS lysate (**C**). Data are displayed as median with 95% confidence intervals. Data points represent individual animals. Statistical tests performed were Kruskal-Wallis with Dunn’s multiple comparisons.

Subunit-vaccinated chickens were tested for their systemic IgY binding against MG lysates of strain R_low_, VA94, or Ap3AS by enzyme-linked immunosorbent assay (ELISA) (Fig. 1). Chickens’ pre-immune sera did not recognize lysates of any MG strain as determined by no significant increase in OD450nm (Fig. 1A through C). Three weeks after the boost vaccination, all subunit-vaccinated chickens recognized each MG lysate as determined by a significant increase in OD450nm (Fig. 1A through C). While the subunit vaccine induced antibody responses reactive against all tested MG strains (Fig. 1A through C), comparative reactivity of anti-subunit antibodies against each MG strain was of interest.

### Individual-subunit-component-vaccinated chickens recognize R_low_, VA94, and Ap3AS lysates through different subunits

**Fig 2:**
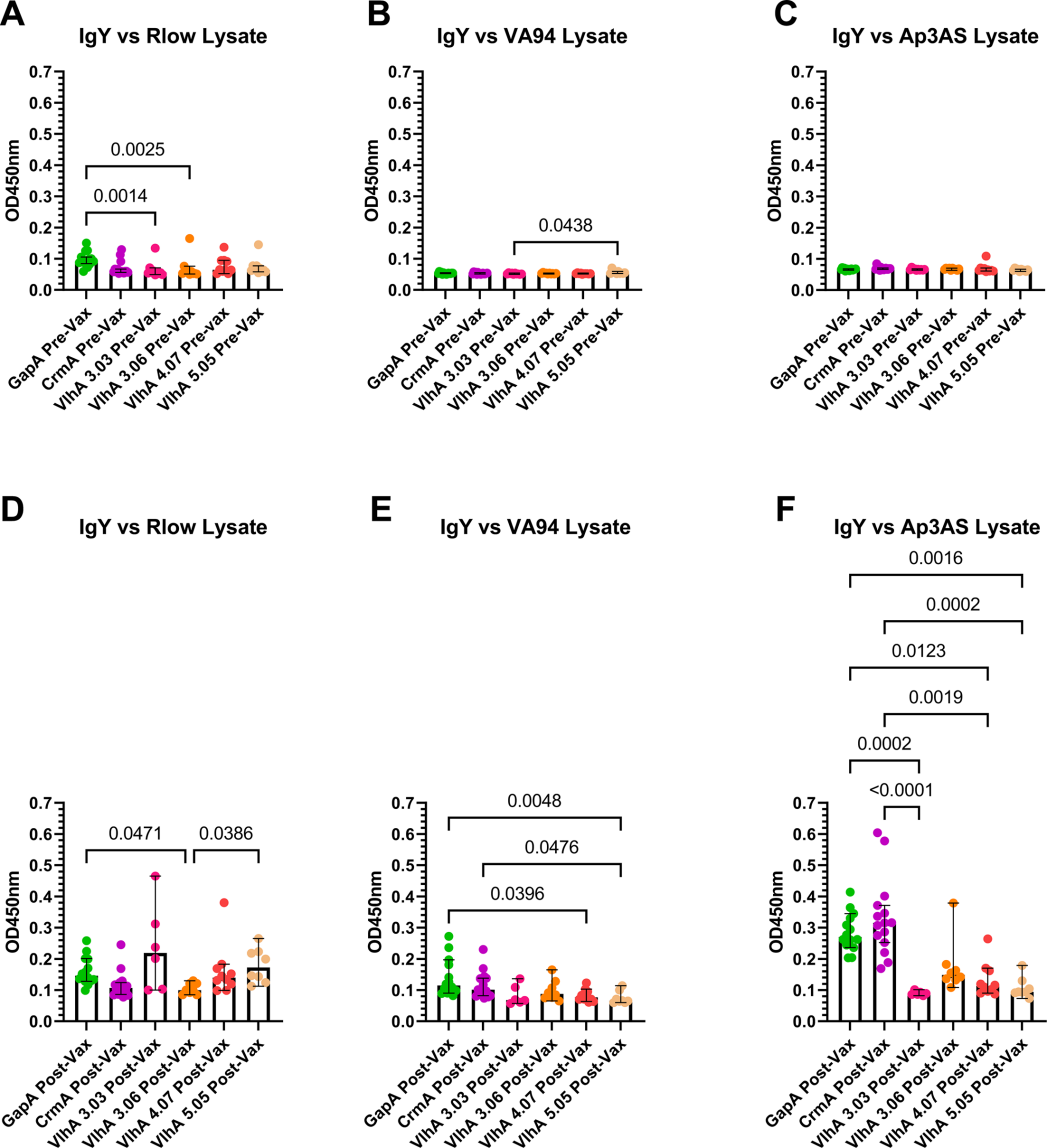
Individual-subunit-component-vaccinated sera vs MG lysates. ELISAs run on sera diluted 1:1,000 from individual-subunit-vaccinated chickens taken prior to vaccination (Pre-Vax) (**A–C**) or taken from chickens 3 weeks post-boost vaccination but prior to challenge (Post-Vax) (**D–F**). Serum IgY was assessed against R_low_ lysate (**A and D**), VA94 lysate (**B and E**), or Ap3AS lysate (**C and F**). Data are displayed as median with 95% confidence intervals. Data points represent individual animals. Statistical tests performed were Kruskal-Wallis with Dunn’s multiple comparisons.

ELISA plates were coated with MG lysates and incubated with sera from individual-subunit-vaccinated chickens (Fig. 2). Recognition of R_low_ lysate, in order from highest to lowest median recognition as determined by higher OD450nm, comes from VlhA 3.03, VlhA 5.05, GapA, VlhA 4.07, CrmA, and VlhA 3.06 (Fig. 2D). Recognition of VA94 lysate, in order from highest to lowest median recognition as determined by higher OD450nm, comes from GapA, CrmA, VlhA 3.06, VlhA 4.07, VlhA 3.03, and VlhA 5.05 (Fig. 2E). Recognition of Ap3AS lysate, in order from highest to lowest median recognition as determined by higher OD450nm, comes from CrmA, GapA, VlhA 3.06, VlhA 4.07, VlhA 5.05, and VlhA 3.03 (Fig. 2F).

### Subunit-vaccinated chickens have reduced disease severity relative to saline-vaccinated chickens when infected with strain VA94

**Fig 3:**
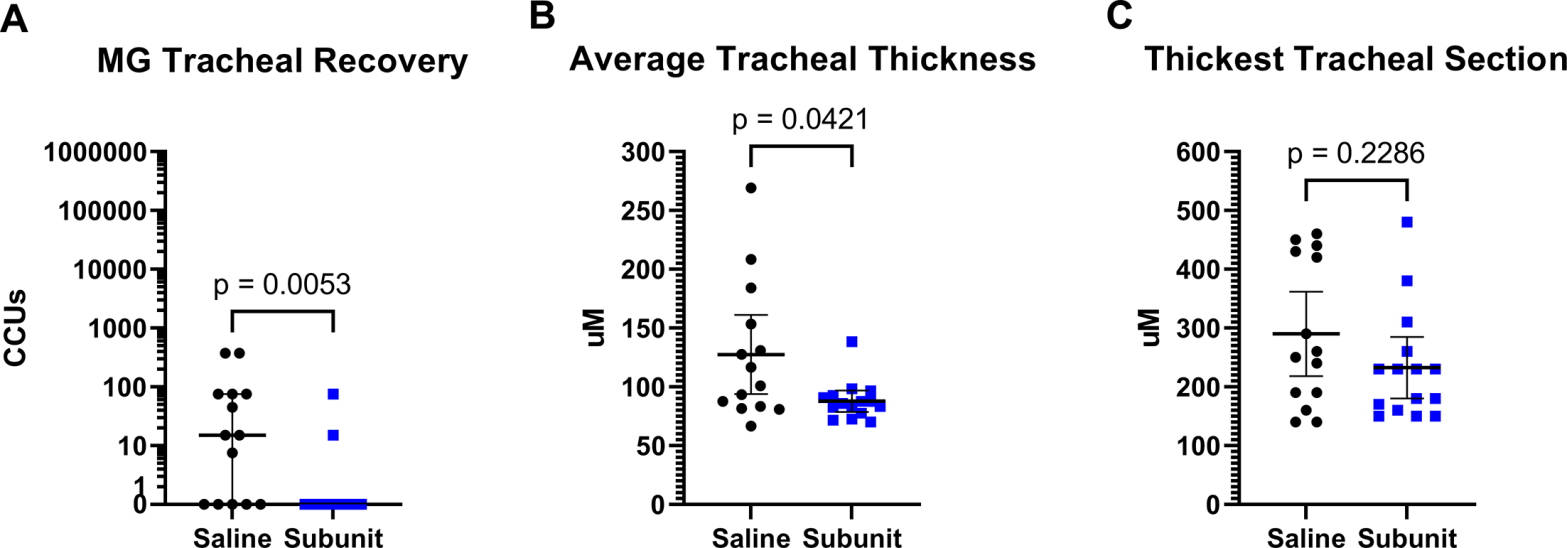
Disease outcomes of chickens infected with MG strain VA94. MG recoveries in color-changing units (CCUs) from chickens that received either saline or the subunit vaccine (**A**). Average tracheal thickness from chickens that received either saline or the subunit vaccine (**B**). Thickest tracheal section from chickens that received either saline or the subunit vaccine (**C**).Panel A is displayed as median with error bars showing the 95% confidence interval. Panels **B and C** are displayed as mean with error bars showing the 95% confidence intervals. Data points represent individual animals. Statistical tests performed were Kruskal-Wallis with Dunn’s multiple comparisons.

The R_low_-based subunit vaccine was examined for protective efficacy against heterologous strain VA94 (Fig. 3). Subunit-vaccinated chickens had statistically significantly reduced MG burden in the trachea (*P* = 0.0053) relative to saline-vaccinated controls (Fig. 3A). Subunit-vaccinated chickens had statistically significantly reduced average tracheal thickness (*P* = 0.0421) relative to saline-vaccinated controls (Fig. 3B) and reduced thickest tracheal sections relative to saline-vaccinated controls, but these later results were not statistically significant (*P* = 0.2286) (Fig. 3C).

### Subunit-vaccinated chickens have reduced disease severity relative to saline-vaccinated chickens when infected with strain Ap3AS

**Fig 4:**
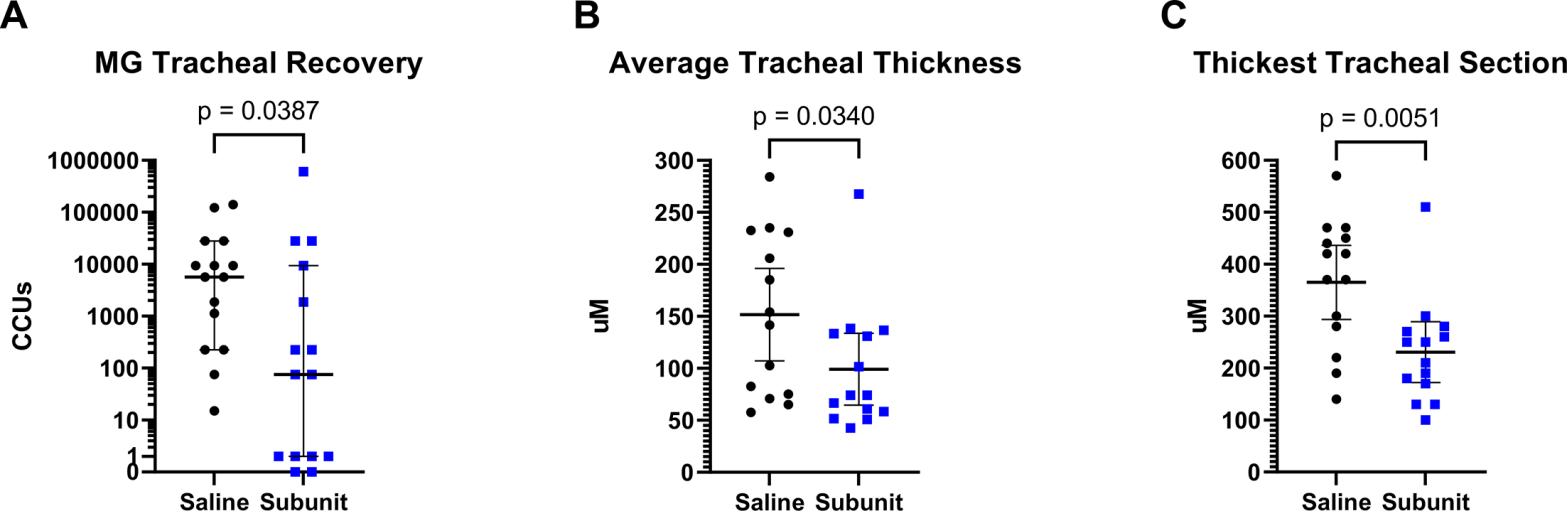
Disease outcomes of chickens infected with MG strain Ap3AS. MG recoveries in color changing units (CCUs) from chickens that received either saline or the subunit vaccine (**A**). Average tracheal thickness from chickens that received either saline or the subunit vaccine (**B**). Thickest tracheal section from chickens that received either saline or the subunit vaccine (**C**).Panel **A **is displayed as median with error bars showing the 95% confidence interval. Panels **B **and **C **are displayed as mean with error bars showing the 95% confidence intervals. Data points represent individual animals. Statistical tests performed were Kruskal-Wallis with Dunn’s multiple comparisons.

The R_low_-based subunit vaccine was examined for protective efficacy against heterologous strain Ap3AS (Fig. 4). Subunit-vaccinated chickens had statistically significantly reduced MG burden in the trachea (*P* = 0.0387) relative to saline-vaccinated controls (Fig. 4A). They also had statistically significantly reduced average tracheal thickness (*P* = 0.0340) relative to saline-vaccinated controls (Fig. 4B), as well as statistically significantly reduced thickest tracheal sections (*P* = 0.0051) relative to saline-vaccinated controls (Fig. 4C).

## DISCUSSION

The R_low_-based MG subunit vaccine demonstrated potential for providing disease severity reductions against multiple heterologous MG strains due to its antigens having similarity to protein identity with tested strains. This was further bolstered by sera from subunit-vaccinated chickens recognizing the lysate of any tested strain (Fig. 1). Cross-reactive potential of the R_low_-specific subunit vaccine was then confirmed through *in-vivo* challenges using strains VA94 and Ap3AS that showed statistically significant reductions in disease severity (Fig. 3 and 4) for subunit-vaccinated chickens.

Although MG strains tend to differ considerably in their VlhA repertoire, some strains still share highly similar individual VlhAs, as shown in Table 1 (28, 43). However, this does not account for which *vlhAs* a particular strain expresses *in vivo*. Previous work has shown that strain R_low_ undergoes *in-vivo vlhA* expression changes through the course of a 7-day infection of the chicken trachea (25). The VlhAs included in the subunit vaccine were designed to cover the breadth of those *vlhAs* expressed during that window of time (19). However, that is a different expression pattern from what is expressed *in vivo* during a 3-day infection of the chicken trachea by VA94. VA94 primarily expresses three *vlhAs* during this time: WP_014886072.1 (HFMG94VA_4667), WP_014886378.1 (HFMGVA_3296), and WP_014886209.1 (HFMGVAA_4650) (26). Thus, although the VlhAs in the subunit vaccine are highly (VlhAs 3.03 and 5.05) or moderately (VlhAs 3.06 and 4.07) similar to VA94 VlhAs (Table 1), this similarity may not be of value during *in-vivo* infection, at least not during the initial days post-exposure, as these are not the *vlhAs* that are actually expressed. Instead, the sequence comparison between the subunit VlhAs and the early-phase *in-vivo*-expressed *vlhAs* of VA94 gives a more realistic picture of the subunit vaccine’s predictive effect (Table 2). These comparisons show that none of the four subunit VlhAs are similar to HFMGVAA_4667, which is of note because this was the most highly expressed *vlhA* in VA94 during the initial days of infection (26). However, all of the subunit VlhAs are somewhat similar to HFMGVA_3296 and HFMGVAA_4650. Strain Ap3AS does not have published data on an early-phase *in-vivo* vlhA expression in chicken tracheas and, thus, cannot be compared in this same way.

The modified GapA and CrmA subunits are highly conserved among these strains (Table 1). Given that GapA is highly similar to both strains and CrmA is nearly identical to both strains, it is likely that if these subunits are contributing to the efficacy of the vaccine, they are providing cross-protection among strains. Because the VlhAs share less sequence identity relative to GapA and CrmA, especially when considering the impact of variable *in-vivo* vlhA expression, if the subunits contribute to protection equally, cross-protection is more likely being driven by GapA and CrmA.

Interestingly, sera from subunit-vaccinated chickens responded more strongly to the Ap3AS lysate than the R_low_ or VA94 lysate (Fig. 1C, A and B, respectively). This is striking given that the antigens in the subunit vaccine were based on protein sequences from strain R_low_. These proteins were produced recombinantly; therefore, conformational similarities could not be assessed. It could be that the recombinant conformations of these proteins are more conducive to eliciting antibodies against epitopes in Ap3AS. It is also possible that Ap3AS has higher expression of the epitopes the antibodies are targeted against. For example, R_low_ has primarily vlhA 3.03 expression when grown in culture (25). Thus, when comparing against MG lysate, most anti-VlhA antibodies from vaccinated chickens will be targeted against this antigen. Strain Ap3AS has primarily WP_318551558.1 expression when grown in culture (G. Browning, personal communication, 2024). When looking at the amino acid level, this VlhA shares 51.72% identity across 12% query cover, 91.67% identity across 5% query cover, 100% identity across 5% query cover, and 91.67% identity across 5% query cover to VlhAs 3.03, 3.06, 4.07, and 5.05, respectively. This shows a large divergence between the subunit VlhAs and the expressed *vlhA* of Ap3AS in culture, suggesting recognition of Ap3AS lysate from subunit-vaccinated chickens is likely coming from anti-GapA and anti-CrmA antibodies rather than anti-VlhA antibodies.

To answer directly what contribution the various subunits had to binding the lysates, we ran individual-subunit-vaccinated chicken sera against R_low_, VA94, and Ap3AS lysates (Fig. 2). The results were in line with our *in-silico* analyses and predictions (Tables 1 and 2). The highest contribution to recognition against R_low_ is from the immunodominant and *in-vitro*-expressed VlhA 3.03 and the highly similar VlhA 5.05 (Fig. 2D); the highest contribution to recognition against VA94 is from GapA and CrmA (Fig. 2E); and the highest contribution to recognition against Ap3AS is from GapA and CrmA (Fig. 2F) (25). Thus, the increased binding to the Ap3AS lysate is primarily driven by the anti-GapA and anti-CrmA antibodies, suggesting higher expression or greater accessibility of these antigens in this strain relative to R_low_.

Reduction in tracheal MG load indicates that the subunit vaccine either induces resistance to initial MG colonization or enhances clearance of established MG. Additionally, reduced MG load suggests a lesser/slower ability to spread the infection (44). The average tracheal thickness is a standard measure used in MG challenge models to assess pathology caused by the MG. This model is used due to its objective nature, simplicity of collection, reproducibility, and translatability across researchers (45). It is also reported as the preferable method for evaluating MG vaccines (46). The thickest tracheal measure is an additional outcome measure implemented by our group in Miller et al. 2024 to improve the overall tracheal pathologic assessment. This measure identifies the single thickest region seen across the measured regions of the trachea and provides the ability to discern between a chicken with infrequent lesions and a chicken with no lesions at all. The thickest tracheal section measurement is useful, as the average tracheal thickness measurement cannot differentiate between those same circumstances.

Although disease severity was reduced against multiple strains, the subunit vaccine may require further optimization before it is ready for commercial use. The vaccine did not entirely prevent MG colonization, and it therefore does not meet the USDA’s National Improvement Plan Standard of using uninfected chickens (7). Although reduced tracheal pathology was a major outcome and has been reported as the preferred method for assessing vaccine efficacy, the effect on egg-laying, hatchability, and feed conversion has yet to be assessed (46). Future work should explore the impact of the vaccine on those outputs.

Overall, the subunit vaccine has shown promise at providing cross-protection against heterologous strains and warrants further development. Given this vaccine’s highly modifiable platform, it may be best utilized by matching antigens to locally circulating strains depending on the region it is used in. Future research should focus on improving the vaccine’s efficacy with a goal of reducing recoverable MG completely. It should also be evaluated regarding transmission and susceptibility in field-like conditions with specific regard to economic outcomes, such as egg production, hatchability, feed conversion, and carcass condemnation along with safety and efficacy evaluations in turkeys.

## MATERIALS AND METHODS

### Studies performed

As previously published in Miller et al., a subunit vaccine adjuvanted with CpG ODN 2007 was utilized for vaccination-challenge studies (19). To generate subunit-vaccinated sera, chickens were vaccinated with either this formulation or saline and used to test the ability of the post-vaccination sera to recognize MG lysates from different strains (*n* = 15). To generate individual-subunit-component-vaccinated sera, chickens were vaccinated with individual components of the subunit vaccine and the CpG ODN 2007 adjuvant (*n* = 15 for GapA or CrmA-vaccinated chickens or *n* = 10 for VlhA 3.03, VlhA 3.06, VlhA 4.07, or VlhA 5.05-vaccinated chickens). These were administered identically as described below with respect to dose, route, and schedule. Heterologous protection data were generated across two studies. The first study compared saline-vaccinated controls to subunit-vaccinated chickens both infected with strain VA94 (*n* = 15). The second study compared saline-vaccinated controls to subunit-vaccinated chickens both infected with strain Ap3AS (*n* = 15). Rarely, data points were removed from/unavailable in the analysis for the following reasons: chicken required euthanasia prior to study termination, the chicken was male, the sample was incorrectly handled/processed/contaminated, or if the chicken did not correctly receive the intended vaccinations.

### Recombinant protein production

Recombinant protein production was performed as described in Miller et al. 2024. In brief, BL21 (DE3) *E. coli* containing expression plasmids containing MG subunit antigens were grown, induced with IPTG, and lysed using bacterial protein extraction reagent (Thermo Fisher, Waltham, MA). His-tagged recombinant subunit proteins were purified from the soluble fraction using HisPur Ni-NTA Spin Columns (Thermo Fisher, Waltham, MA). Total and specific protein quantities were estimated using Qubit 4 Fluorometer (Thermo Fisher, Waltham, MA) and SDS-PAGE with densitometry (Image Lab, Bio-Rad).

### Vaccine formulations and preparations

All subunit vaccines were formulated using 50 µg of each recombinant MG subunit protein and adjuvanted with 25 µg CpG ODN 2007. Components were combined, and physiological saline was added to obtain a final volume of 500 µL per dose.

### Inoculum preparation

MG was grown at 37°C for 24 h in Hayflick’s medium to achieve log-phase growth. MG was then centrifuged at 10,000 × g at 4°C for 10 min. The supernatant was removed, fresh medium was added, and the bacteria were resuspended. OD620 readings were taken to determine estimated CFU. MG concentration was adjusted to 5 × 10^8^ CFU/mL in Hayflick’s medium. Inocula were placed on ice until the challenge. After the infection, the inoculum was assayed to confirm viable titer of challenge.

### Infection studies

Animal studies were approved and performed in accordance with our Institutional Animal Care and Use Committee (IACUC) Protocols (A22-008 and A25-003). Four-week-old specific pathogen-free female white leghorn chickens (AVS Bio, Norwich, CT) were randomly divided into pens upon arrival. Five-week-old chickens were leg banded for identification. The next day, chickens were primarily vaccinated subcutaneously between the back and right wing. Eight-week-old chickens were boost vaccinated identically as before. At 11 weeks on Day 0, chickens were intratracheally infected with 200-µL Hayflick’s medium containing 1 × 10^8^ estimated CFU of MG strain VA94 or MG strain Ap3AS. CFU were determined by OD620. Chickens were infected identically on Day 2 with the same strain used on Day 0. On Day 14, chickens were necropsied by the administration of Telazol (Covetrus, Portland, ME) at a dose of 10 mg/kg followed by cervical dislocation. Tracheas were excised, and excess tissue was trimmed from the trachea. Three total sections coming from the proximal, middle, and distal regions of the trachea were taken for bacterial recovery (1 cm) and histology (<0.5 cm). Sections for recovery were put into 3-mL Hayflick’s medium and placed on ice. Sections for histology were put into 5 mL of 10% neutral buffered formalin.

### Blood collection and heat-inactivated sera preparation

In brief, blood was collected from the wing vein at 5 weeks old prior to vaccination and at 11 weeks old post-boost vaccination but pre-challenge. Blood was allowed to clot for 1 h. It was then centrifuged at 1,000 × g for 10 min at room temperature. Sera were pipetted off into a tube and heated at 56°C for 30 min. Sera were then frozen at −20°C for future use (19).

### Histology and tracheal thickness

Tracheal sections were allowed to fix for 24–48 h before being trimmed and submitted to the Connecticut Veterinary Medical Diagnostic Laboratory (CVMDL) for embedding, sectioning, and hematoxylin and eosin staining. Slides were evaluated in a blinded manner, and tracheal measurements encompassing the mucosa and submucosa were taken. Each tracheal ring (three rings per sample) was measured at 12, 3, 6, and 9 o’clock. These measurements were averaged to determine average tracheal thickness. Additionally, the largest measurement that could be found anywhere across all three rings per sample was taken as the thickest tracheal section.

### Bacterial recovery

After necropsy, tracheal sections that were put on ice in Hayflick’s medium were vortexed four times for 30 s. Samples were then incubated at 37°C for 3 h. Samples were then put through a 0.45-µM Cytiva Whatman Uniflo syringe filter (Cytiva Life Sciences, Marlborough, MA) and 5-fold serially diluted in duplicate in Hayflick’s medium. Growth was determined by color change (acidification) from red to yellow. Duplicate wells were averaged to determine the bacterial recovery by color changing units (CCUs).

### Enzyme-linked immunosorbent assays (ELISAs)

Maxisorp ELISA plates (Thermo Fisher Scientific, Agawam, MA) were coated at 37°C for 1 h with 0.5 µg of MG lysate per well in 100 µL of carbonate/bicarbonate buffer. Plates were then washed three times using phosphate-buffered saline with 0.05% (vol/vol) tween-20 (PBS-T). Plates were then blocked with 3% (wt/vol) bovine serum albumin in PBS-T for 1 h. Plates were then washed three times with PBS-T. A quantity of 100 µL of desired primary antibody sample (sera diluted 1:1,000 into PBS-T) was loaded into each well and incubated at room temperature for 1 h. Plates were then washed three times with PBS-T. A quantity of 100 µL of secondary antibodies, HRP-conjugated goat anti-chicken IgY (Sigma-Aldrich, Saint Louis, MO) diluted 1:30,000 in PBS-T was loaded into each well and incubated at room temperature for 1 h. Plates were then washed three times with PBS-T. Plates were then developed by loading 100 µL of 1-Step Ultra TMB ELISA Substrate (Thermo Fisher Scientific, Agawam, MA) into each well and incubating at room temp for 3 min and 30 s. The reaction was quenched by adding 100 µL of ELISA Stop Solution (Thermo Fisher Scientific, Agawam, MA) per well. Optical densities were then read at 450 nm using a Cytation 5 (Agilent Biotek, Winooski, VT) plate reader.

### Protein alignment comparisons

Protein alignments were performed using National Center for Biotechnology Information (NCBI) BLASTP. Sequences for subunit proteins were derived from strain R_low_ using NCBI reference sequence NC_004829.2. VA94 sequences were derived from NCBI reference sequence NC_018406.1. Ap3AS sequences were derived from NCBI reference sequence NZ_CP044624.1.

### Data analyses

ELISAs, bacterial recoveries, average tracheal thickness, and thickest tracheal section were analyzed in GraphPad Prism 9. ELISAs were analyzed by Kruskal-Wallis with Dunn’s multiple comparisons test. All other analyses were performed using Mann-Whitney U tests.
